# In-Vitro Inhibition of Staphylococcal Pathogenesis by Witch-Hazel and Green Tea Extracts

**DOI:** 10.3390/antibiotics8040244

**Published:** 2019-11-29

**Authors:** Reuven Rasooly, Adel Molnar, Hwang-Yong Choi, Paula Do, Kenneth Racicot, Emmanouil Apostolidis

**Affiliations:** 1 U.S. Department of Agriculture, Agricultural Research Service, Albany, CA 94710, USA; paula.do@ars.usda.gov; 2 Department of Chemistry and Food Science, Framingham State University, Framingham, MA 01702, USA; adelmmolnar@gmail.com (A.M.); kelolo123123@gmail.com (H.-Y.C.); 3 United States Army Combat Capabilities Development Command—Soldier Center (CCDC-SC), Natick, MA 01760, USA; racicot.civ@mail.mil

**Keywords:** Staphylococcal Enterotoxin A (SEA), biofilm inhibition, Hamamelitannin (HAMA), *Staphylococcus aureus*, *Staphylococcus epidermidis*

## Abstract

whISOBAX (WH), an extract of the witch-hazel plant that is native to the Northeast coast of the United States, contains significant amounts of a phenolic compound, Hamamelitannin (HAMA). Green tea (GT) is a widely consumed plant that contains various catechins. Both plants have been associated with antimicrobial effects. In this study we test the effects of these two plant extracts on the pathogenesis of staphylococci, and evaluate their effects on bacterial growth, biofilm formation, and toxin production. Our observations show that both extracts have antimicrobial effects against both strains of *S. aureus* and *S. epidermidis* tested, and that this inhibitory effect is synergistic. Also, we confirmed that this inhibitory effect does not depend on HAMA, but rather on other phenolic compounds present in WH and GT. In terms of biofilm inhibition, only WH exhibited an effect and the observed anti-biofilm effect was HAMA-depended. Finally, among the tested extracts, only WH exhibited an effect against Staphylococcal Enterotoxin A (SEA) production and this effect correlated to the HAMA present in WH. Our results suggest that GT and WH in combination can enhance the antimicrobial effects against staphylococci. However, only WH can control biofilm development and SEA production, due to the presence of HAMA. This study provides the initial rationale for the development of natural antimicrobials, to protect from staphylococcal colonization, infection, or contamination.

## 1. Introduction

Staphylococci are gram-positive bacteria that can cause multiple diseases, from minor skin infections to severe device associated infections, sepsis, and death. Staphylococcal species like *S. aureus*, which are coagulase positive bacteria, cause diseases through the production of multiple toxins, and antibiotic resistant strains like MRSA (methicillin resistant *S. aureus*) are commonly found [1,2,3]. Staph species like *S. epidermidis* belong to the coagulase negative staphylococcal (CNS) group and cause disease mostly through the formation of biofilms that are highly resistant to antimicrobials and to the host’s immune defenses [4,5].

Staphylococcal species, including *S. auerus* and *S. epidermidis*, are part of the healthy normal microflora of the skin and mucus membranes. However, they become pathogenic when their numbers increase, and they reach a certain quorum [2,6]. Quorum sensing systems are then activated, leading to virulence (e.g., production of toxins and/or formation of biofilms) [7,8]. These quorum-sensing systems also allow the bacteria to survive under oxidative stress conditions that are part of the host’s immune response, thus allowing the bacteria to better survive in the host [9,10].

There are multiple ways to prevent or treat bacterial infections. These include enhancing the host’s immune response through vaccination, or interfering with bacterial survival by the use of antibiotics that e.g., disrupt bacterial cell walls or interfere with their replication. But even with the advancement of multiple therapeutic approaches now available, these bacteria cause millions of deaths around the world. The World Health Organization ranks bacterial resistance to antibiotics as one of the top three health care concerns worldwide, with staphylococcal bacterial infections being the largest contributors to this growing problem. In the US, according to the Center for Disease Control (CDC), of the estimated annual two million hospital-acquired infections in the US, approximately one half is due to staph bacteria. Annually, these infections result in over 90,000 deaths and over 100,000 amputations. Furthermore, antibiotic use can lead to disruption of the normal microflora, potentially giving rise to other health issues [11,12,13,14,15]. Alternatives to standard antibiotic treatment are thus needed, considering both genetic resistance as well as behavioral resistance (through formation of biofilms) [7,11,15]. 

Plants contain a large range of phenolic compounds of various polarities. These compounds are important because of their scavenging abilities due to their abundant levels of hydroxyl groups [16]. Based on some structural differences, polyphenols are subdivided into several major subclasses—phenolic acids, stilbenes, tannins, diferuloylmethanes, and flavonoids. These are potent antioxidants, and have multiple biological effects, including antimicrobial activity [17,18,19]. For example, green tea plant extracts containing high quantities of carvacrol, epicatechin, and epicatechin gallate polyphenols suppress bacterial growth in food [20]. The antibacterial activity of polyphenols has been attributed to their nonspecific hydrogen bonding and hydrophobic effects to microbial cell wall, membranes, adhesion molecules, enzymes, and cell envelope transport proteins [20,21,22]. For example, EGCG (epigallocatechin gallate) has been shown to bind peptidoglycan layers, thus disrupting their function as protective layers, making bacteria more vulnerable to environmental changes. Gallic Acid has been shown to interact with bacterial lipid bilayers (of both of gram-positive and gram-negative bacteria), thus disrupting cell function by increasing cell permeability, as well as interfering with cell adhesion, motility, sporulation, and spreading [21,23]. Tannic Acid was shown to interact with microbial enzymes, thus interfering with cellular metabolism and function. Gallotannins have been shown to chelate iron, which is vital for the survival of most pathogenic bacteria. Tannic acid and EGCG have been shown to specifically interfere with AHL-mediated quorum sensing signaling in gram-negative bacteria, leading to disruption of various quorum-sensing mediated functions like swarming motility [23,24]. Hamamelitannin has been shown to act as a quorum sensing inhibitor of staphylococci, inhibiting biofilms from forming and toxins from being produced [24,25,26,27,28].

Our aim is to use plant extracts rich in polyphenols that would limit the ability of the bacteria to evade host’s immune response, while interfering with their ability to produce toxins or form biofilms. The extracts tested were witch-hazel and green tea, because of their known antibacterial properties. Green tea extracts are rich in flavanols and their gallic acid derivatives, like catechin, epicatechin (EC), gallocatechin (GC), epicatechin gallate (ECG), epigallocatechin (EGC), and the most abundant one, epigallocatechin gallate (EGCG) [29]. Both catechins and gallates have been shown to have antibacterial properties due to their interaction with the bacterial cell wall, proteins and/or membrane phospholipids, causing increased cell permeability, inhibiting respiration, and altering ion transport processes [30,31,32,33].

Witch-hazel (*Hamamelis virginiana*) bark contains high levels of phenolic compounds like Hamamelitannin (HAMA, 2’,5-di-O-galloyl-d-hamamelose), which is a low molecular weight tannin. HAMA has been shown to act as a quorum sensing inhibitor in staphylococci, inhibiting bacterial virulence (toxin production and biofilm formation) [27]. Witch-hazel also contains other phenolic compounds, such as gallic acid, gallocatechin, and epigallocatechin, which cause bacterial cell disruption that result from binding to bacterial membranes [21,34].

In this paper, we tested the phenolic content of witch-hazel extract rich in HAMA (whISOBAX (WH), StaphOff Biotech Inc., Hopkinton, MA USA) and green tea extract (GT) (Naturex, Avignon, France) and compared their antimicrobial activity against planktonic and biofilm bacteria. The extracts were tested on *S. epidermidis*, a bacteria notorious for their ability to form biofilms and are common causes of device-associated infections. The extracts were also tested on *S. aureus*, a bacteria notorious for their antibiotic resistance and for their ability to cause sepsis due to the multiple toxins they can produce. 

## 2. Results

### 2.1. Determination of Total Phenolic Content of WH and GT

The total phenolic content of WH and GT was tested. For WH (whISOBAX, a 50 mg/mL solution), the phenolic content was determined to be 12.66 mg/mL gallic acid equivalent (GAE). For a green tea 10 mg/mL solution the phenolic content was determined to be 10 mg/mL GAE, which is not surprising, since the extract purchased by Naturex has been standardized to a phenolic content > 98%. To understand how much of the WH phenolic content was due to HAMA, the total phenolic content (as GAE) of a 1 mg/mL of HAMA standard was determined to yield 0.544 mg/mL GAE. With this in mind, and knowing that the HAMA content in the extract is 17.3 mg/mL (as quantified by HPLC (High Pressure Liquid Chromatography) see below), we can expect that out of the 12.66 mg/mL GAE phenolic in the extract, 9.41 mg/mL GAE is due to HAMA (75%) and the remaining 3.25 mg/mL GAE is due to other phenolic compounds naturally present in the witch-hazel extract, like gallic acid, gallocatechin, and catechin [27].

### 2.2. Determination of HAMA Content in WH by HPLC

**Figure 1:**
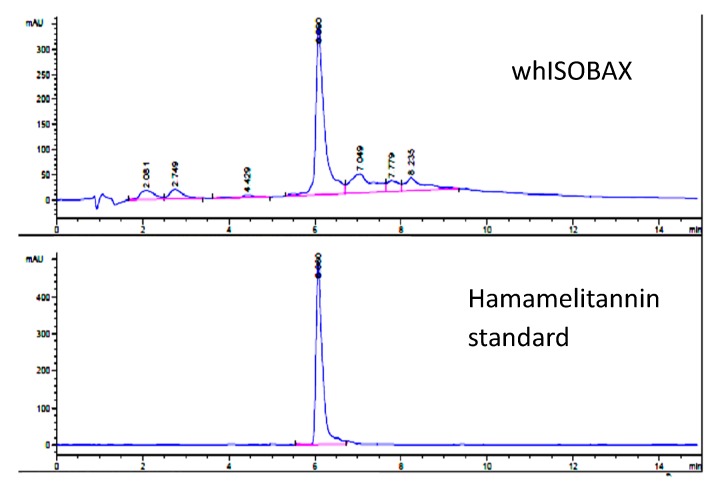
Determination of hamamelitannin in whISOBAX (WH) by reverse phase HPLC analysis. WH (StaphOff Biotech Inc) or HAMA (Sigma-Aldrich) were applied to Durashell reverse phase C18 column in water containing 0.1% trifluoroacetic acid (TFA). Bound material was eluted with an acetonitrile gradient. The amount of HAMA in WH was determined by comparing the retention time and absorbance spectrum with the HAMA standard.

WH was analyzed by reverse phase HPLC and HAMA content was determined by comparison with a HAMA standard (Sigma-Aldrich). As shown in Figure 1, a single primary peak is evident at 210 nm, which was determined to be HAMA by comparison of absorbance profile to a HAMA standard and confirmed by liquid chromatography mass spectrometry (LCMS) analysis. The amount of HAMA in WH was calculated as 17.3 mg/mL. 

### 2.3. Antibacterial Activity Against Planktonic Cells

**Figure 2:**
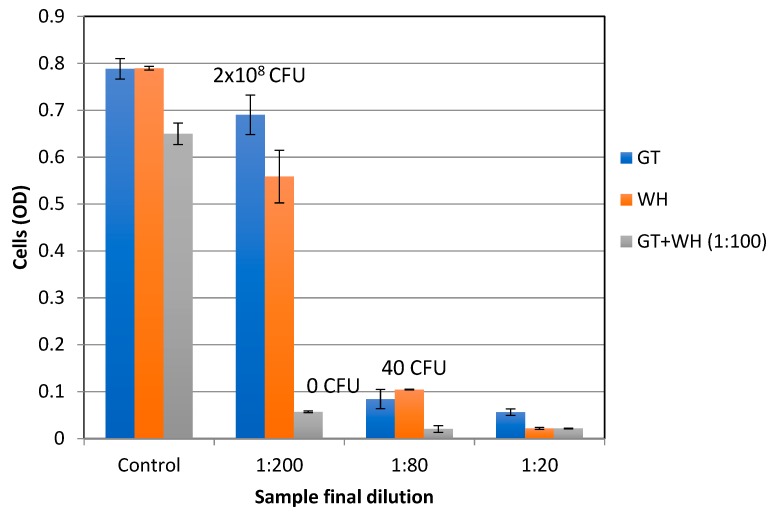
The effect of GT and WH on the growth of *S. epidermidis*. Bacteria were grown overnight at 37 °C with increasing concentrations of GT, WH, or GT with WH diluted 1:100, and cell density determined spectrophotometrically at OD_630_. The control solution for GT or WH was TSB (Tryptic Soy Broth) alone, while control solution for GT+WH 1:100 was WH 1:100. Cells were plated and CFU (colony-forming units) indicated.

**Figure 3:**
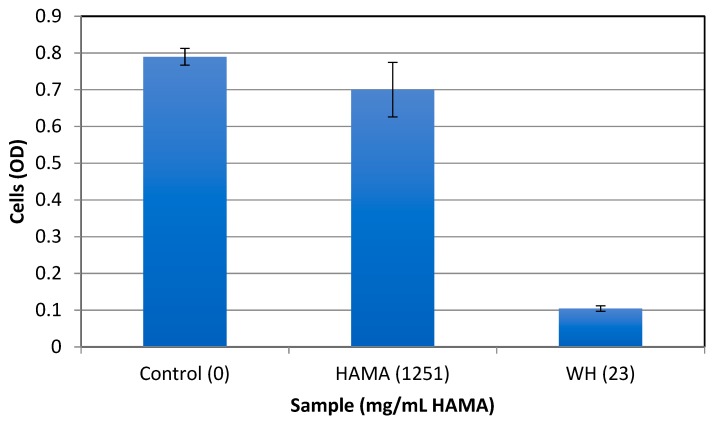
The effect of HAMA on the growth of *S. epidermidis*. Bacteria were grown overnight at 37 °C with HAMA (Sigma-Aldrich, 1251 mg/mL) or with WH (StaphOff Biotech Inc) diluted 1:80 (containing 23 mg/mL HAMA), and cell density determined spectrophotometrically at OD_630_. As a control, cells were grown in TSB only.

To test for the antibacterial activity of the extracts, early exponential *S. epidermidis* cells were grown overnight with increasing extract concentrations, and the MIC (Minimal inhibitory concentration) and MBC (minimal bactericidal concentration) were determined using spectrophotometric and plating methods. The stock solutions of GT (10 mg/mL) and WH (50 mg/mL) that were used had a phenolic content of 10 mg/mL GAE and 12.66 mg/mL GAE, respectively. The stock solutions were evaluated at various dilutions (0 to 2000 times diluted). Table 1 shows the phenolic and dry weight content of GT and WH in the tested dilutions. As shown in Figure 2, the MBC of WH and GT was determined to be at 1:40 dilutions, which corresponds to 0.31 mg/mL GAE and 0.25 mg/mL GAE, respectively. The MIC was observed at 1:80 dilutions, which corresponds to phenolic contents of 0.125 mg/mL GAE for GT and 0.15 mg/mL GAE for WH. At the MBC level of WH, the amount of HAMA content is 0.23 mg/mL. As previously reported (e.g., [25]), when HAMA was tested alone, even at higher concentrations of over 50 times more than its content in effective WH concentrations, HAMA did not have any antibacterial effect (Figure 3), suggesting that the antibacterial effect of WH is due to other phenolic compounds present, like gallic acid, gallocatechin, and catechin [34].

To evaluate the possible synergistic antibacterial effect of GT and WH on the growth of *S. epidermidis*, GT was tested at the same doses with or without a single sub-inhibitory dose of WH (1:100 dilution that corresponds to 0.126 mg/mL GAE). As shown in Figure 2, in the presence of WH diluted 1:100, the MBC of GT significantly decreased (*p* < 0.01) by 5-fold, from 1:40 to 1:200 (from 0.25 to 0.05 mg/mL GAE). 

**Figure 4:**
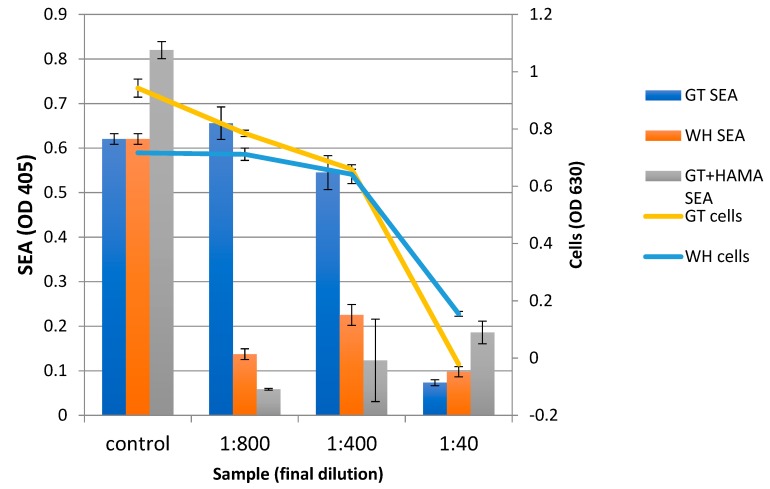
The effect of GT, WH, or HAMA on *S. aureus* growth and toxin production; *S. aureus* cells were grown overnight with increasing concentrations of GT or WH, or with increasing concentrations of GT+0.043 mg/mL HAMA. Cell density was measured (Cells), cells removed by centrifugation, and the presence of SEA was determined in cell supernatants by enzyme-linked immunosorbent assay (ELISA) (SEA).

The antibacterial effect of GT and WH were tested also on *S. aureus*. Bacteria were grown overnight with increasing concentrations of GT or WH. As shown in Figure 4, the MIC for both GT and WH was observed at 1:80 dilutions, which correspond to a phenolic content of 0.125 mg/mL GAE and 0.157 mg/mL for GT and WH, respectively. The MBC for both treatments was observed at the 1:40 dilution, that corresponds to a phenolic content of 0.25 mg/mL GAE and 0.214 mg/mL GAE for GT and WH, respectively. Importantly, GT and WH were also shown to inhibit the growth of the MRSA strain *S. aureus* ATCC 43300, where their MICs were ~0.03 mg/ml GAE [35].

### 2.4. The Effect of WH and GT on Staphylococcal Pathogenesis (Biofilm Formation and Toxin Production)

The hallmark of *S. aureus* pathogenesis is the production of multiple toxins that are highly regulated by quorum sensing systems and are produced only when the bacteria reaches a certain cell density. One of those toxins is Staphylococcal Enterotoxin A (SEA), which belongs to a family of heat stable enterotoxins that act as super-antigens and are a leading cause of gastroenteritis resulting from consumption of contaminated food [36].

The dose-dependent effect of WH and GT was tested on *S. aureus* SEA production by ELISA (Figure 4). In the presence of GT, the amount of SEA produced paralleled the cell growth pattern (Figure 4), i.e., more toxins were produced as more cells were present, suggesting that GT had no effect on SEA production. More specifically, we only observed a reduction of SEA production at the 1:40 dilution of GT, which is its MBC against *S. aureus* (Figure 4). On the other hand, SEA production was inhibited in the presence of WH, even at concentrations that did not inhibit cell growth. More specifically, with WH, we observed a reduced SEA production at the 1:800 dilution of WH (0.015 mg/mL GAE) while the MBC of WH was observed at the 1:40 dilution (0.214 mg/mL GAE). The inhibitory effect of WH on SEA production is most likely due to its high HAMA content, as HAMA has been shown to suppress toxin production in *S. aureus* [27]. Indeed, when HAMA and GT were combined, SEA production diminished, and their inhibition profile was similar to that of WH (Figure 4). Of note is that the amount of HAMA (0.043 mg/mL) was tested because it represented a concentration of HAMA that is found in WH 1:400, without having any effect on cell growth. 

**Figure 5:**
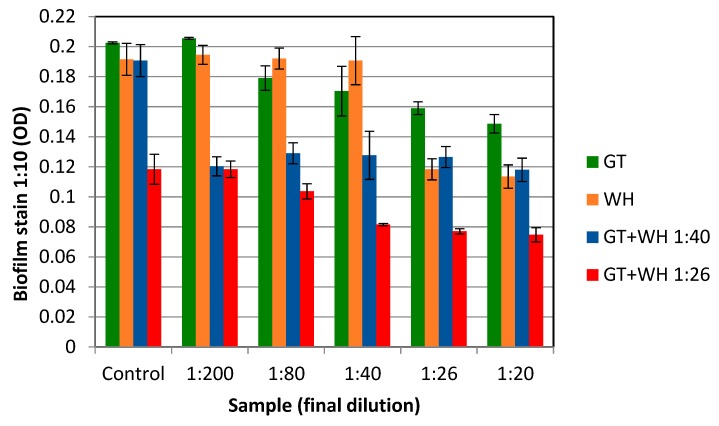
The effect of GT and WH on pre-formed *S. epidermidis* biofilm; Cells were grown in microtiter plates with slight shaking for 4 hrs. Unbound cells were removed and bound cells (biofilm cells) were further incubated overnight with increasing concentrations of GT, WH, or a combination of the two extracts. Unbound cells were removed. Remaining attached (biofilm) bacteria were washed and stained with crystal violet, and their OD determined. As a control for single extract treatment of GT or WH, cells were grown in TSB alone. As a control for combination treatments of GT+WH, cells were grown with no GT but with WH at 1:26 or 1:40 dilutions.

The hallmark of *S. epidermidis* pathogenesis is the formation of biofilms, which are highly resistant to antibiotic therapy. The effect of WH and GT on eradicating *S. epidermidis* biofilms was tested by first forming biofilms, consisting of about 1 × 10^7^ CFU. These biofilms were then exposed to various doses of GT, WH, or GT+WH for 12 h (to about 1 × 10^9^ CFU if untreated). We observed that GT had a minimal inhibitory effect on *S. epidermidis* biofilms (Figure 5), even when tested at higher concentrations of the observed MBC against *S. epidermidis*. WH, on the other hand, was more effective against biofilm bacteria, reducing biofilm load to almost 50% when at the 1:26 dilution (0.47 mg/mL GAE). The inhibitory effect of WH on bacterial biofilms is likely due to its high HAMA content [24,27].

To test for the possible synergistic effect of the two extracts, increasing concentrations of GT were mixed with two WH dilutions, 1:40 (0.31 mg/mL GAE) and 1:26 (0.47 mg/mL GAE). These two WH doses were selected because when WH was tested alone we observed no biofilm inhibition at the 1:40 dilution, and the 1:26 dilution was the first dilution that an observed effect (Figure 5). As shown in Figure 5, when GT was mixed with the two WH dilutions, an enhanced biofilm inhibitory effect was observed. More specifically, even the 1:40 WH dilution, when combined with GT, resulted in a significant biofilm reduction also at the lowest GT doses tested (Figure 5). Higher (1:26) WH dilutions in combination with various concentrations of GT resulted in an even greater biofilm reduction (Figure 5) (*p* < 0.01). 

## 3. Discussion

The results presented here indicate that GT suppresses staphylococcal growth while WH suppresses both staphylococcal growth and pathogenesis (Biofilm formation and toxin production). These factors are important in acute infections (planktonic-associated) and in chronic infections (biofilm-associated). The results presented here also show that WH and GT are synergistic to one another and enhance their respective antibacterial activities. 

The inhibitory effect of WH on the growth of both *S. epidermidis* and *S. aureus* was tested and the MBC/MIC against planktonic cells was determined to be at 0.31/0.15 mg/mL GAE (Figure 2 and Figure 4). The MIC of WH needed to inhibit a biofilm was 3× higher, at 0.47 mg/mL GAE (Figure 5). This is not surprising, considering that biofilms are known for their enhanced tolerance to antibacterial treatments. While biofilm cells were more tolerant to the inhibitory effect of WH, tolerance was reduced when the two extracts were combined. WH is very effective at inhibiting S. aureus from producing SEA, and is shown to suppress toxin production even at low concentrations of 0.015 mg/mL GAE (Figure 4). 

The effect of GT against growth of planktonic cells (*S. aureus* or *S. epidermidis*) was tested, and its MBC/MIC was shown to be 0.125/0.25 mg/mL GAE (Figure 2 and Figure 4). However, GT had a minimal effect on biofilm reduction, even at the highest tested dose of 0.5 mg/mL GAE (Figure 5). GT also had no effect on *S. aureus* SEA production, since any observed reduction in SEA production was only due to the direct inhibition of GT on *S. aureus* growth (Figure 4). 

WH or GT alone did not have any significant effect on biofilm growth while the same concentration caused a reduction in SEA production. This further indicates that while some phenolic compounds in both GT and WH affect cell growth, other phenolic compounds that are only present in WH affects toxin production. This compound was shown to be Hamamelitannin; At the MBC level of WH against planktonic cells (0.31 mg/mL GAE), the amount of HAMA content is 0.23 mg/mL. HAMA alone, even at 20-fold higher concentrations, had no effect on bacterial growth, suggesting that the antibacterial effect of WH is not due to its HAMA content but due to other phenolic compounds present, like gallic acid, gallocatechin, and catechin [34]. On the other hand, HAMA was an important factor in suppressing bacterial virulence (biofilm by *S. epidermidis* and toxin production by *S. aureus*), as GT alone had no effect on bacterial virulence, but inhibitory activity was observed when mixed with WH or HAMA (Figure 4 and Figure 5). While we could not show any effect of GT alone on staphylococcal quorum-sensing (QS) mediated functions such as biofilm formation or toxin production, its anti-QS activity had been demonstrated in gram-negative bacteria [37]. 

Studies on the molecular mechanism of HAMA indicate that it inhibits bacterial pathogenesis (biofilm formation and toxin production) by interfering with QS systems that are necessary for the bacteria to survive within the host [6,9,10]. QS is a communication system between bacteria, which are activated by chemicals secreted by the bacteria itself that in turn activate signal transduction pathways, leading to regulation of genes that are necessary for bacterial survival once their numbers increase and a quorum is reached. In *S. aureus*, those include activation genes encoding for toxins, like surface proteins that promote colonization of host tissues, invasins (leukocidin, proteases, hyaluronidase) that promote the spread of bacteria in tissues; membrane-damaging toxins (hemolysins, leukotoxin, leukocidin) that puncture human cell membranes, thereby causing cell damage and/or death; and exotoxins staphylococcal enterotoxins, toxin shock syndrome toxins (SEs, TSST) that damage host tissues and cause symptoms of disease like fever, inflammation, low blood pressure, circulatory collapse, and death [2]. Collectively inhibiting the production of the many toxins by HAMA-rich WH would greatly benefit the host [2,27]. 

HAMA inhibits staphylococcal *agr/TraP* quorum sensing regulatory systems, leading to a change in the expression of multiple genes important for cell survival and virulence (stress response, toxin production, and biofilm formation) [10,27]. HAMA has also been shown to affect *S. aureus* biofilm susceptibility to different classes of antibiotics (through the TraP receptor), by affecting cell wall synthesis [24]. Bacteria are then unable to overcome the stressors they encounter during infections, and thus become more vulnerable to the host’s immune response and to antibiotics. 

The antibacterial activity of witch-hazel can be seen as a two-throng approach, where some of the phenolic compounds act to disrupt bacterial cells, reducing their number. At the same time, HAMA disrupts residual biofilm cells while also preventing toxin production, thus inhibiting cells from causing harm to the host. Collectively, these specific phenolic compounds hinder bacterial survival in the host, allowing eradication of both acute and chronic (biofilm-based) infections. The addition of green tea, with its strong antibacterial activity, complements that of WH, further enhancing their respective antibacterial activity.

In conclusion, the results presented here clearly indicate that WH is very effective in suppressing both growth and virulence of coagulase negative and coagulase positive staphylococci, while GT is very effective in suppressing only planktonic cell growth. Our results also indicate the benefit of using a combination of WH and GT for the suppression of staphylococcal pathogenesis, with the synergist effects of the anti-bacterial properties exhibited by GT and WH, along with the strong anti-biofilm and anti-toxin production exhibited by WH. Findings from this work provide the basic biochemical rationale for the further evaluation of witch-hazel and green tea for the development of natural remedies to staph-associated infections and contaminations.

## 4. Materials and Methods 

### 4.1. Bacteria

*S. epidermidis strain* ATCC 35984 (RP62A), a biofilm producing strain and *S. aureus* USDA strain, an enterotoxin A producer., were used for this study. The bacteria were grown in Tryptic Soy Broth (TSB) with shaking (220 RPM) at 37 °C overnight, diluted 1:500 in TSB, and grown for about two more hours to the early exponential phase of growth of about 0.1 OD_630_.

### 4.2. Test Formulations

whISOBAX (WH) was supplied by StaphOff Biotech Inc. (Hopkinton, MA, USA) and a polyphenol standardized (> 98% phenolic content) green tea extract (GT) was supplied by Naturex (Avignon, France). Unless noted, all chemicals were purchased from Sigma-Aldrich Co. (St. Louis, MO, USA). 

### 4.3. Total Phenolic Content Determination for whISOBAX

The total phenolic content was determined essentially as described [31]. Briefly, 0.5 mL of the sample (WH, GT, HAMA, or increasing concentrations of Gallic Acid standard) was mixed with 0.5 mL distilled water, 1 mL 95% ethanol, 5 mL distilled water, and 0.5 mL 50% (v/v) Folin–Ciocalteu reagent, and incubated at 22 °C for 5 min. One milliliter of 5% Na_2_CO_3_ was added, and the mixtures were kept in the dark at 22 °C for 1hr. The solution was mixed by vortexing, and the absorbance was determined spectophotometrically at 725 nm. The results were expressed as mg of gallic acid equivalents (GAE) per gram of sample of dried extract weight (DW) or per sample volume. The data presented are an average of three measurements.

### 4.4. Hamamelitannin Content in WH (HPLC Determination)

WH was analyzed by High Pressure Liquid Chromatography (HPLC) and the HAMA content was determined by comparison to a standard HAMA sample, according to Wang et al. [34] with some modifications to provide a faster method that is less susceptible to solvent composition, and is compatible with LC requirements. The column used was the Durashell reverse phase C18 (Agilent Technologies, Santa Clara, CA, USA) 3 µm, 100 Ǻ, 4.6 x 50 mm column. The solvents used were acetonitrile/water (both containing 0.1% TFA) gradient. HPLC (Agilent 1200 System, Agilent Technology, Santa Clara, CA, USA) was used with a variable wavelength Detector. The amount of HAMA in WH was confirmed by comparing the retention time and absorbance spectrum with the HAMA standard. LCMS analysis (Agilent 1100 System, Agilent Technology, Santa Clara, CA, USA was carried out using the detector stmospheric pressure chemical ionization in mass spectrometry (APCI-MS), in positive mode (carried out by Organix Inc. Woburn, MA, USA).

### 4.5. Antibacterial Testing on Planktonic Cells

The minimal inhibitory concentration (MIC) was determined using a microbroth dilution method with an initial inoculum of early exponential bacteria. All test dilutions were made in TSB, to obtain a similar GAE content between test extracts (Table 1). Specifically, cells were grown to the early exponential phase of growth in TSB and cells (20 µL, approximately 2 × 10^4^ CFU per well) were incubated with increasing dilutions of test solutions in a final volume of 200 µL per well (Polystyrene 96-well plates (Falcon, Corning, NY, USA)) for about 18 h at 37 °C in air. The cell density was determined using a microtiter plate reader (BioTek, Winooski, VT, USA) at an optical density of 600 nm or 630 nm. The cell number was determined by plating samples on Tryptic Soy Agar (TSA) plates, incubating overnight at 37 °C, and colony-forming units (CFU) counted the next day. The MIC was taken as the lowest drug concentration resulting in observable colonies. The minimal bactericidal concentration (MBC) was taken as the lowest drug concentration that resulted in no observable colonies. All experiments were performed in triplicates. The optical density (OD) of test solutions in TSB (no cells) were determined and used as background values. The positive controls included growing cells in TBS alone or TSB with relevant solvents. 

### 4.6. Antibacterial Testing on Biofilm Cells

The biofilm assays were carried out essentially as described [17,38,39]. Bacteria were grown in TSB to their early exponential phase of growth (OD_630_ of about 0.045, which was about 1000 CFU/µL). To develop a biofilm, 200 µL were placed in 96 polystyrene well plates (Falcon, Corning, NY, USA), and grown for 4–5 h with gentle agitation (~50 RPM) at 37 °C. Unbound cells were removed, and bound cells were rinsed two times with sterile Phosphate Buffer Saline (PBS) under aseptic conditions. (Sample wells were fixed with ethanol to determine initial biofilm by staining (see below)). To adherent cells (approximately 6 × 10^6^ CFU), 200 µL test solutions (in TSB) were added, and the microtiter plates were incubated for ~18 h at 37 °C with gentle agitation (50 RPM). The cell density was determined spectophotometrically at OD_630_. Non-adherent cells (“cells”) were removed to another microtiter plate and the cell density was determined. CFU was determined by plating a sample on TSA plates.

To evaluate the formation of a biofilm, the remaining attached bacteria (“biofilm”) were washed three times with PBS, fixed with ethanol, ethanol was then removed, and the cells were air-dried. Biofilm cells were then stained for 5 min with filtered 0.2% crystal violet in 20% ethanol. The unbound stain was rinsed off with water. The plates were air-dried and the dye bound to adherent cells was solubilized with 200 µL 0.1% sodium dodecyl sulfate (SDS). The OD of each well was determined at 630 nm (BioTek Microplate Reader, Winooski, VT, USA). The tests were performed in triplicates. 

### 4.7. Staphylococcal Enterotoxin A (SEA) Production 

“Sandwich” ELISA testing was used to determine the amount of SEA produced by *S. aureus* as described [36]. Specifically, sheep anti-SEA IgG (Toxin Technology, Sarasota, FL, USA) was used as the capture antibody, and sheep anti-SEA Horse Radish Peroxidase (HRPO) (Toxin Technology, Sarasota, FL, USA) was used as the detection antibody. The capture antibody was diluted in a coating buffer (0.01 M NaHCO_3_, 0.1 M Na_2_CO_3_) at a final concentration of 10 µg/mL and 100 µL/well was added to microtiter 96-well plates (Costar, Washington D.C., USA) and incubated for 1hr at 37 °C or overnight at 4 °C. The plates were washed three times with PBST (PBS containing 0.05% Tween-20), and the same solution (100 µL/well) was used for blocking unbound sites for 15 min at room temperature (RT). To prepare test samples, the treated cells were removed by centrifugation, and the supernatants were collected. One hundred microliters of each sample were added (in triplicate wells) and the plates were incubated for 2 h at 37 °C. The plates were washed three times with PBST. A detection antibody, diluted 1:300 in PBST, was added (100 µL/well), and incubated for 1 h at 37 °C. The plates were washed five times with PBST. One hundred microliters of 3,3’,5,5;-tetramethylbenzidine chromogen solution (Invitrogen, Carlsbad, CA, USA) substrate was added, and 0.3 HCl (50 µL/well) was added to stop the reaction. The absorbance was measured at 450 nm in a microplate reader (BioTek, Winooski, VT, USA) and expressed as 10X OD measured. All tests were performed in triplicate. Increasing amounts of SEA (1 µg/mL to 10 ng/mL) was used as a standard curve.

### 4.8. Statistical Analysis

All experiments were carried out in triplicates and the averages were presented. The standard deviation was calculated using the “unbiased” n-1 method by Microsoft Excel. The significance of differences between treatment groups was calculated using a two-tailed Student’s t-test. *p* < 0.05 was considered significant.

## 5. Practical Applications

The high content of phenolic compounds in Green Tea (GT) and the high content of hamamelitannin in whISOBAX (WH) make these products ideal for restoring oral and digestive health, and enhancing food safety and stability. The synergist effects of the anti-bacterial properties exhibited by GT and WH, along with the strong anti-biofilm and anti-toxin production exhibited by WH, support the development of nutraceutical alternatives to antibiotics, to enhance food-safety and health.

## Figures and Tables

**Table 1:** Phenolic content of GT and WH at tested dilutions.

Final Dilution of Tested Extracts	GT Phenolic Content (mg/mL GAE)	WH Phenolic Content (mg/mL GAE)
1:2000	0.005	0.006
1:800	0.012	0.015
1:400	0.025	0.031
1:200	0.050	0.063
1:80	0.125	0.157
1:40	0.250	0.315
1:26	0.375	0.471
1:20	0.500	0.630
1:16	0.625	0.790
